# Spinopelvic Alignment as an Associated Factor of Short-Term Diagnostic Response to Lumbar Medial Branch Block: A Prospective Study

**DOI:** 10.3390/jcm15114354

**Published:** 2026-06-04

**Authors:** Burcu Ozalp, Argun Pire, Gonul Sari, Meltem Uyar, Can Eyigor, Gunay Yolcu

**Affiliations:** 1 Department of Algology, Faculty of Medicine, Ege University, Izmir 35100, Turkey; argunp@gmail.com (A.P.); meltemuyar@gmail.com (M.U.); caneyigor@yahoo.com.tr (C.E.); 2 Department of Algology, Faculty of Medicine, Adnan Menderes University, Aydın 09100, Turkey; drgonulsari@gmail.com; 3 Department of Physiotherapy and Rehabilitation, Faculty of Medicine, Celal Bayar University, Manisa 45030, Turkey; dryolcugunay@gmail.com

**Keywords:** chronic low back pain, facet joint, medial branch block, spinopelvic alignment, pelvic incidence, lumbar lordosis

## Abstract

**Background**: Lumbar facet joints are a significant source of chronic low back pain (CLBP), and medial branch blocks (MBBs) are the widely accepted reference diagnostic approach for diagnosis. However, clinical response varies. This study aims to investigate whether sagittal spinopelvic alignment parameters can predict the clinical efficacy of MBB in patients with facet-mediated CLBP. **Methods**: In this prospective observational study, 110 patients (aged 40–80) with facet-related CLBP underwent diagnostic MBBs using a double-block protocol. Spinopelvic parameters, including pelvic incidence (PI), pelvic tilt (PT), sacral slope (SS), and lumbar lordosis (LL), were measured on standing lateral radiographs. Clinical response was defined as a ≥80% reduction in Visual Analog Scale (VAS) scores. Data were analyzed using multivariate logistic regression and Receiver Operating Characteristic (ROC) curves. **Results**: Responders (*n* = 68) were significantly younger and had a lower BMI than non-responders (*n* = 42) (*p* < 0.05). Non-responders exhibited significantly higher PI–LL mismatch (18.6° ± 7.4 vs. 3.9° ± 4.2, *p* < 0.001), higher PT (23.6° ± 5.1 vs. 17.4° ± 4.5, *p* < 0.001), and lower LL (35.8° ± 7.2 vs. 45.2° ± 6.4, *p* < 0.001). ROC analysis identified a PI–LL mismatch threshold of >12.5° as the strongly associated with negative short-term diagnostic response (AUC = 0.892). Multivariate analysis confirmed that PI–LL mismatch > 12.5° was a potential associated factor within the investigated model of poor response (OR: 4.25, 95% CI: 2.10–8.60, *p* < 0.001), while age and BMI were not significant in the adjusted model. **Conclusions**: Sagittal spinopelvic malalignment, specifically an increased PI–LL mismatch, is strongly associated with reduced diagnostic utility of MBB. Integrating biomechanical assessment into clinical decision-making may improve patient selection and treatment outcomes for facet-mediated pain.

## 1. Introduction

Chronic low back pain (CLBP), recognized as the leading cause of functional disability worldwide, represents a pathophysiologically heterogeneous spectrum. Among the potential nociceptive sources, the lumbar facet joints play a pivotal role and have been reported as the primary pain generator in approximately 15–45% of patients with chronic lumbosacral symptoms [1]. Facet joint pain is highly sensitive to disturbances in the biomechanical load distribution of the spinal column. In this context, afferent signaling transmitted via the medial branch nerves is central to the persistence and chronification of pain perception. Contemporary clinical guidelines classify medial branch blocks (MBBs) as the “Widely accepted reference approach” for both the diagnosis of facet-mediated pain and its minimally invasive management, serving not only as a therapeutic intervention but also as a reference diagnostic approach [2].

Recent biomechanical paradigms evaluate spinal pathologies within the broader context of global spinopelvic balance (sagittal alignment), rather than as isolated segmental degenerations [3]. Spinopelvic architecture reflects a dynamic interplay between pelvic morphological parameters—such as pelvic incidence (PI), pelvic tilt (PT), and sacral slope (SS)—and global alignment components including lumbar lordosis (LL) and sagittal vertical axis (SVA). Emerging evidence highlights that disruption of this balance leads to a posterior shift of the gravitational axis, resulting in an “axial overload” phenomenon affecting the facet joints [4,5].

Current literature suggests that sagittal malalignment—particularly PI–LL mismatch and increased SVA—reduces the load-bearing capacity of the intervertebral discs, thereby transferring mechanical stress to posterior spinal elements [6]. This chronic mechanical burden is thought to induce microvascular damage, synovial hypertrophy, and neurogenic inflammation within the facet joints. However, the variability in clinical response to facet medial branch block (MBB) has not yet been adequately explored in relation to baseline spinopelvic geometry in prospective settings [7,8].

While previous literature, such as Park et al., has heavily relied on structural, qualitative advanced imaging to identify MRI-based associated factors of MBB response, there remains a critical knowledge gap regarding how global, forces-driven spinal alignment influences treatment outcomes. To address this, the present study explicitly contrasts traditional structural imaging by focusing on quantitative, whole-spine biomechanical parameters, exploring the clinical relevance of sagittal alignment characteristics in this patient population [7].

While most existing studies focus on structural degeneration or pain severity, the present study aims to investigate whether global biomechanical alignment parameters may serve as associated factors of response to interventional treatment outcomes. To the best of our knowledge, this is the first prospective study evaluating the relationship between sagittal spinopelvic alignment and clinical response to lumbar medial branch block in patients with facet joint-related chronic low back pain.

This prospective study aims to assess the relationship between individual spinopelvic profiles and changes in VAS scores before and after intervention and to evaluate the independent association of these factors with short-term diagnostic response.

## 2. Materials and Methods

This study was designed as a prospective observational investigation to evaluate the relationship between spinopelvic configuration and clinical outcomes following facet joint medial branch block in patients with chronic low back pain of facet origin. The study was conducted at the Algology Clinic of Ege University Faculty of Medicine after obtaining ethical approval from the Non-Interventional Clinical Research Ethics Committee of Izmir Bakircay University (Decision No: 1615, Date: 30 May 2024). All procedures were carried out in accordance with standard clinical follow-up protocols of the institution.

Patients aged between 40 and 80 years who presented to the pain clinic with low back pain and met the inclusion criteria were enrolled in the study. Eligible participants had a history of chronic low back pain persisting for at least three months and demonstrated insufficient response to conservative treatment modalities.

Exclusion criteria included the presence of concomitant conditions that could account for lumbar pain (such as rheumatologic diseases, infection, trauma, or major spinal fractures), prior spinal surgery, previous treatment with interventional pain procedures, coexisting psychiatric disorders or limited cooperation, and morbid obesity (body mass index > 50 kg/m^2^). Patients meeting any of these criteria were excluded from the study.

Data were collected systematically during outpatient visits through clinical assessments, diagnostic investigations, and pre- and post-procedural scoring. Demographic variables—including age, sex, and body mass index—were recorded using a standardized data collection form and prepared for statistical analysis.

An open-label design was adopted, whereby patients were aware of the intervention being performed. To minimize measurement bias, all evaluation procedures were standardized. The physicians performing the intervention, assessing pain outcomes, and the clinicians responsible for measuring spinopelvic parameters were assigned independently. Pain assessments were conducted by a separate researcher using the VAS, while radiological measurements were performed by another experienced physician. This approach was intended to reduce observer bias and enhance data objectivity.

Pain intensity was assessed using the VAS during movement-evoked conditions (specifically during lumbar extension and walking), as facet-mediated pain is characteristically exacerbated by mechanical loading. Patients were asked to indicate their pain level on a 10-cm scale, where 0 corresponded to “no pain” and 10 to “worst imaginable pain.” All VAS scores were recorded in a standardized data collection form. Measurements were obtained at two time points: at baseline during the outpatient visit (VAS-1) and following the facet joint medial branch block (VAS-2). Each time point was analyzed separately in the statistical evaluation.

To confirm facet joint-mediated pain, diagnostic lumbar MBBs were performed. The medial branch blocks were performed either unilaterally or bilaterally, strictly depending on the clinical distribution of the patient’s symptoms (unilateral vs. bilateral low back pain). The interventions targeted the medial branches corresponding to the symptomatic levels, most frequently involving the L3, L4 and L5 medial branches to anesthetize the L3–L4, L4–L5 and L5–S1 facet joints. Crucially, no sedation or systemic analgesics were administered to the patients prior to or during the procedures, as sedation can impair cognitive feedback and confound the immediate post-block pain assessment.

All procedures were carried out under sterile conditions by an experienced interventional pain specialist. Patients were positioned prone on the fluoroscopy table, and target levels were determined based on clinical findings and magnetic resonance imaging. Following antiseptic skin preparation, the anatomical course of the medial branch nerves innervating the relevant facet joints was identified under fluoroscopic guidance (OEC, GE Healthcare, Chicago, IL, USA). Target points were defined at the junction of the superior articular process and the transverse process. After local skin anesthesia, a 22-gauge spinal needle (Egemen International, Izmir, Turkey) was advanced under fluoroscopic guidance to the target site, and correct positioning was confirmed using both anteroposterior and oblique views. After negative aspiration to exclude intravascular placement, 0.5 mL of 1% lidocaine was injected at each medial branch. To enhance diagnostic accuracy and reduce false-positive results, a comparative double-block protocol was employed on a separate day using a long-acting local anesthetic, 0.5% bupivacaine (0.5 mL per level). Pain intensity was reassessed at predefined intervals following each procedure using the VAS. Patients who demonstrated a dynamic pain reduction of at least 80% during the acute post-block diagnostic window (assessed at 30 min for lidocaine, and at 1 h for bupivacaine) following both the first diagnostic block with lidocaine and the second confirmatory block with bupivacaine were classified as ‘Responders’ (positive short-term diagnostic response). Conversely, patients who exhibited a positive response to only one of the local anesthetics or showed no significant pain relief after either procedure were classified as ‘Non-responders’ (negative short-term diagnostic response). A positive diagnostic response was defined as a ≥80% reduction in pain intensity consistent with the expected duration of action of the anesthetic agents [9,10].

The selection of a stringent ≥ 80% pain reduction threshold—rather than a more permissive ≥50% cut-off—was strictly aligned with the guideline-concordant dual-block protocols recommended by the Spine Intervention Society (SIS) and the American Society of Interventional Pain Physicians (ASIPP). Utilizing an 80% threshold is widely accepted as the clinical reference standard to rigorously mitigate false-positive rates inherent to single blocks, isolate true facet-mediated nociception, and optimize patient selection for subsequent thermal radiofrequency neurotomy [11,12].

**Figure 1:**
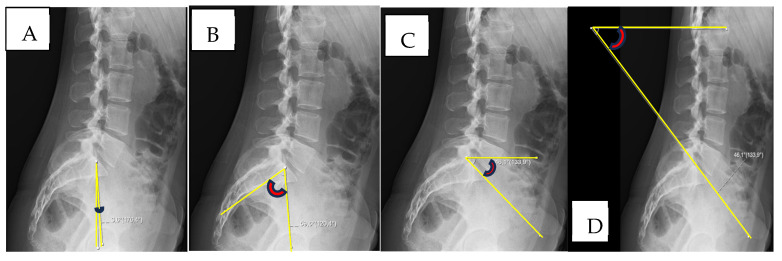
Measurement of Pelvic Incidence (PI), Pelvic Tilt (PT), Sacral Slope (SS), and Lumbar Lordosis (LL). Schematic representation of sagittal spinopelvic parameters measured on a standing lateral radiograph. Pelvic incidence (PI) (**A**) is defined as the angle between a line perpendicular to the sacral endplate at its midpoint and a line connecting this point to the femoral head axis. Sacral slope (SS) (**B**) is the angle between the sacral endplate and the horizontal plane. Pelvic tilt (PT) (**C**) is defined as the angle between the vertical axis and the line connecting the femoral head axis to the midpoint of the sacral endplate. Lumbar lordosis (LL) (**D**) is measured using the Cobb method between the superior endplates of L1 and S1. PI–LL mismatch is calculated as the difference between pelvic incidence and lumbar lordosis.

Spinopelvic parameters were measured independently by two experienced specialists. Measurements were performed on standardized lateral radiographs using digital measurement software (Centricity PACS, Version 6.0; GE Healthcare, Chicago, IL, USA). Each observer evaluated pelvic incidence (PI), pelvic tilt (PT), sacral slope (SS), and lumbar lordosis (LL) independently and in a blinded manner. Interobserver agreement was assessed by comparing measurement results statistically. In cases of significant discrepancy, parameters were re-evaluated jointly until consensus was reached. This approach was implemented to enhance measurement reliability and reproducibility. The angular measurements for pelvic incidence, pelvic tilt, sacral slope, and lumbar lordosis are shown in Figure 1.

## 3. Statistical Analysis

Statistical analyses were performed using IBM SPSS Statistics software (Version 26.0; IBM Corp., Armonk, NY, USA), with internal validation modules executed using Python (Version 3.10; Python Software Foundation, Wilmington, DE, USA) utilizing scikit-learn packages (Version 1.2). Continuous variables were expressed as the mean ± standard deviation, while categorical variables were presented as counts and percentages (%). Between-group comparisons were conducted using the independent samples *t*-test for continuous variables and the chi-square test for categorical variables. Factors associated with treatment response were evaluated using multivariate logistic regression analysis. Variables that displayed a *p*-value of <0.10 in the bivariate analysis (Table 1 and Table 2), including age, BMI, and PI–LL mismatch, were entered into the multivariate model. Baseline VAS and sacral slope were excluded due to a lack of statistical significance in the univariate screening (*p* > 0.05). Global spinopelvic parameters (PI–LL mismatch, PT, and LL) were analyzed both as continuous variables (with Odds Ratios calculated per 5° increase) and as dichotomous categories based on ROC-derived cut-off thresholds. The diagnostic performance of parameters was assessed using receiver operating characteristic (ROC) curve analysis and the Youden index.

To evaluate internal validity and address potential optimism bias in the multivariate regression configuration, the discriminatory performance of spinopelvic parameters for short-term diagnostic response was validated using both Bootstrapping (with 1000 resamples) and 5-fold Cross-Validation. The optimism-corrected Area Under the ROC Curve (AUC) along with its 95% Confidence Interval (CI) was calculated to provide a robust estimate of the model’s performance.

Sample size adequacy was evaluated using a priori power analysis. Based on the primary outcome measure (PI–LL mismatch), assuming 80% power and α = 0.05, a minimum sample size of approximately 26 patients per group was calculated for a moderate effect size (Cohen’s d ≈ 0.5). A post hoc power analysis was also conducted. Using the observed mean ± standard deviation values of responders and non-responders (3.9 ± 4.2 vs. 18.6 ± 7.4), Cohen’s d was calculated as approximately 2.44. Given this effect size, the total sample size (*n* = 110) was sufficient to detect significant differences between groups and to support multivariate analyses. However, given the study design, these characteristics should be interpreted within the study context, and further external validation is warranted.

Interobserver reliability for radiographic spinopelvic parameters was evaluated using the Intraclass Correlation Coefficient (ICC) with a 95% confidence interval based on a two-way random-effects model with absolute agreement.

## 4. Results

A total of 128 patients were initially evaluated. Of these, 8 declined participation and 10 were excluded due to the absence of suitable lateral radiographs for spinopelvic measurements. Eventually, 110 patients were included in the final analysis. The demographic and baseline clinical characteristics of the study population are summarized in Table 1. No major adverse events or complications, including infection, hematoma formation, or transient motor blockade, were observed in any of the patients throughout the study period.

When comparing patients who demonstrated a significant response to facet block (*n* = 68) with those who did not (*n* = 42), responders were found to be significantly younger (64.2 ± 10.5 vs. 73.1 ± 8.9 years, *p* = 0.001) and had lower body mass index (29.1 ± 3.6 vs. 32.2 ± 4.1 kg/m^2^, *p* = 0.012). No statistically significant differences were observed between groups in terms of sex distribution or baseline VAS scores (*p* > 0.05). Post-treatment VAS scores, however, showed a marked reduction in the responder group compared to non-responders (2.4 ± 0.9 vs. 5.1 ± 1.2, *p* < 0.001).

The interobserver reliability for radiographic measurements was excellent, with ICC values ranging from 0.86 to 0.92 for all spinopelvic parameters. Radiographic spinal alignment parameters measured from standing lateral radiographs are presented in Table 2. Notably, PI–LL mismatch was significantly higher in the non-responder group and emerged as a strong factor associated with short-term diagnostic response within the investigated model.

In subgroup analyses, patients were stratified based on age and body mass index into two categories: younger patients with lower BMI (age < 65 years and BMI < 30) and older patients with higher BMI (age > 65 years or BMI > 30). In the younger/lower BMI subgroup, PI–LL mismatch was significantly lower in responders compared to non-responders (2.8° ± 3.1 vs. 12.4° ± 5.2, *p* = 0.003). Similarly, pelvic tilt (PT) values were lower in responders (16.1° ± 3.4 vs. 21.8° ± 4.1, *p* = 0.008).

A comparable pattern was observed in the older/higher BMI subgroup. Responders exhibited significantly lower PI–LL mismatch values than non-responders (4.2° ± 4.8 vs. 20.1° ± 8.3, *p* < 0.001). Pelvic tilt was also significantly lower among responders (18.5° ± 5.2 vs. 24.9° ± 5.9, *p* = 0.001). These subgroup findings are detailed in Table 3.

Receiver operating characteristic (ROC) curve analysis was performed to evaluate the discriminatory performance of spinopelvic parameters in identifying patients with a negative short-term diagnostic response. A PI–LL mismatch threshold of >12.5° demonstrated the highest diagnostic accuracy for identifying poor response, indicating robust descriptive performance. The initial multivariate logistic regression model combining baseline demographics and global spinopelvic parameters demonstrated a high descriptive AUC. However, after adjusting for potential optimism bias through rigorous internal validation, the 5-fold cross-validated AUC settled at a robust 0.828. Furthermore, bootstrapping with 1000 resamples demonstrated an optimism-corrected AUC of 0.831 (95% CI: 0.754–0.908) for PI–LL mismatch, confirming the stability and internal validity of the investigated configuration on unseen clinical data. ROC analysis parameters are summarized in Table 4.

In the continuous evaluation of multivariate logistic regression, every 5° increase in baseline PI–LL mismatch was associated with a significant 1.74-fold increase in the odds of exhibiting a negative short-term diagnostic response (OR = 1.74, 95% CI: 1.28–2.36, *p* < 0.001).

Subsequently, when the ROC-derived threshold was applied to the multivariate configuration as a categorical variable, a PI–LL mismatch > 12.5° remained the strongest factor associated with short-term diagnostic response within the investigated model, demonstrating a 4.25-fold increased risk of a negative short-term diagnostic response (OR: 4.25, 95% CI: 2.10–8.60, *p* < 0.001) after fully controlling for age and BMI. In contrast, neither continuous nor categorical evaluations of age and BMI retained statistical significance in the adjusted multivariate regression model (Table 5).

## 5. Discussion

The most notable finding of this study is the demonstration of a significant relationship between sagittal spinopelvic alignment and short-term diagnostic response to lumbar medial branch block (MBB). While previous studies have largely focused on the association between spinopelvic parameters and structural degeneration or pain severity, our findings extend this perspective by suggesting that global sagittal alignment may also be associated with the acute outcomes of interventional diagnostic procedures.

To our knowledge, this is the first prospective study to evaluate the relationship between sagittal spinopelvic alignment and short-term diagnostic response to MBB. Our results indicate that non-responders exhibit significantly greater PI–LL mismatch, increased pelvic tilt, and reduced lumbar lordosis. Among these parameters, PI–LL mismatch emerged as the most discriminative factor within the investigated model, underscoring the potential role of sagittal imbalance in modulating diagnostic outcomes.

From a biomechanical standpoint, an increased PI–LL mismatch reflects a loss of harmony between pelvic morphology and lumbar curvature, which compromises sagittal balance [13]. This imbalance often triggers compensatory pelvic retroversion, manifested as increased pelvic tilt, to maintain an upright posture [14]. However, such compensation shifts mechanical loading toward the posterior spinal elements, particularly the facet joints [15].

Chronic overload of the facet joints may lead to capsular strain, synovial irritation, and persistent nociceptive signaling via the medial branch nerves [16,17]. In this context, even if an MBB temporarily interrupts nociceptive transmission, the underlying biomechanical stress persists. This persistent mechanical burden may limit the overall short-term diagnostic response, providing a plausible explanation for the inadequate pain reduction observed in patients with pronounced sagittal imbalance.

Several underlying pathophysiological factors can contribute to an increased PI–LL mismatch, including osteoporotic vertebral compression fractures, advanced degenerative disc disease, and paraspinal muscle atrophy (such as in the multifidus and erector spinae muscles). A reduction in muscle strength weakens the posterior tension band of the spine, accelerating sagittal malalignment. While our study establishes an association between PI–LL mismatch and a negative short-term diagnostic response, we did not directly evaluate paraspinal muscle volume or the presence of subtle vertebral fractures. These factors could concurrently drive both the worsening of sagittal balance and the chronicity of mechanical back pain, representing a crucial area for future mechanistic research.

Our findings are consistent with previous literature linking spinopelvic parameters to degenerative changes and pain. Prior studies have shown that higher pelvic incidence and altered pelvic tilt are associated with increased mechanical stress on lower lumbar facet joints [18]. This finding aligns with our results, where higher PI and PT values were observed in the non-responder group, reinforcing the role of sagittal alignment in modulating mechanical loading of the facet joints. Similarly, PI–LL mismatch has been correlated with both pain severity and functional disability in patients with chronic low back pain, as demonstrated by Sarkar et al. using Oswestry Disability Index (ODI) scores [19].

Interestingly, previous studies examining the relationship between sagittal alignment and interventional responses have yielded inconsistent results. For example, investigations in geriatric populations reported no significant association between PI–LL mismatch and response to epidural steroid injections [20]. This discrepancy may be explained by differences in target pain generators. While epidural injections primarily target radicular inflammation, MBBs directly address facet-mediated nociception. Consequently, the influence of spinopelvic alignment may be more pronounced in conditions directly involving the posterior spinal elements.

Importantly, our study evaluates these principal spinopelvic parameters using both continuous scales and ROC-derived thresholds within multivariate models. The consistency of PI–LL mismatch across both evaluations suggests a potential dose–response relationship within our study cohort, where the odds of presenting a negative short-term diagnostic response escalate by 1.74-fold for every 5° increase in baseline mismatch. This indicates that subtler degrees of sagittal malalignment may incrementally alter global spinopelvic loading.

Furthermore, the relationship between sagittal imbalance and diagnostic response persisted independently of age and BMI in our adjusted multivariate model. While these preliminary findings suggest that sagittal spinopelvic alignment plays a clinically meaningful role in the context of short-term diagnostic block response, they should be interpreted cautiously. Given the exploratory nature of our dataset, these results should be viewed as hypothesis generating rather than a definitive protocol for patient selection or a clinical management pathway. Pre-procedural assessment of spinopelvic alignment may simply help clinicians temper prognostic expectations and identify patients who might benefit from earlier multimodal strategies, such as rehabilitation programs targeting sagittal balance alongside routine interventional diagnostic screening.

## 6. Limitations

Several limitations should be acknowledged. First, this study was conducted at a single tertiary center with a relatively small cohort of 110 patients, which may limit the generalizability of our results to broader chronic low back pain (CLBP) populations. Second, despite utilizing a guideline-concordant, comparative dual-block protocol with a stringent 80% pain reduction threshold, the potential for placebo effects or false-positive responses cannot be completely eliminated in clinical pain research.

Third, our multivariate analysis relied on global spinopelvic metrics and basic demographics. CLBP is a complex, multidimensional syndrome influenced by various unmeasured clinical, functional, and radiographic variables. We did not account for the exact grade of facet arthropathy on MRI, baseline functional disability scores, symptom duration, concomitant medication use, physical rehabilitation history, or psychosocial confounders. Furthermore, advanced structural and muscular drivers of sagittal malalignment—such as paraspinal muscle atrophy or fatty infiltration—were not quantitatively evaluated. Consequently, the observed association between PI–LL mismatch and short-term diagnostic response should be interpreted cautiously and viewed as a potential marker within this study configuration rather than an isolated, definitive biomarker.

Additionally, spinopelvic parameters derived from standing lateral radiographs represent a static, two-dimensional projection of a dynamic, three-dimensional system. This approach does not capture segmental asymmetry, rotational deformities, coronal imbalance, or dynamic, movement-evoked loading variations. Because standing postures can be affected by acute pain, lower limb alignment, or fatigue, some alignment differences in non-responders might reflect temporary antalgic adaptations rather than permanent structural geometry.

Finally, our cohort was predominantly female and older, which represents a population prone to accelerated disc degeneration and sarcopenia. While age- and BMI-stratified subgroup analyses demonstrated that the association of PI–LL mismatch remained consistent, sex-specific biomechanical variations cannot be completely ruled out. Due to the observational nature of this study, definitive conclusions regarding causality cannot be made.

While these preliminary findings suggest that a baseline PI–LL mismatch greater than 12.5° is associated with a negative short-term diagnostic response, this threshold should not be used as an absolute exclusion criterion for medial branch blocks. Instead, it may serve as an early, hypothesis-generating indicator to temper prognostic expectations. In patients with pronounced sagittal malalignment, clinicians might consider contextualizing the diagnostic block as a short-term assessment while concurrently encouraging earlier conservative, balance-focused physical therapy. Future prospective, multicenter trials with larger, matched cohorts are required to validate these observations.

## 7. Conclusions

In conclusion, our study suggests that sagittal spinopelvic malalignment, particularly an increased PI–LL mismatch, is significantly associated with a lower likelihood of a positive short-term diagnostic response to lumbar medial branch blocks. These findings highlight the potential value of considering global sagittal profiles alongside traditional structural pathology during clinical evaluations. However, given the single-center design, static 2D imaging approach, and absence of external validation, these results remain exploratory and hypothesis generating. Rather than serving as strict exclusion criteria, pre-procedural spinopelvic assessment may help clinicians risk-stratify patients and prompt further research into integrated biomechanical and interventional strategies for chronic low back pain.

## Figures and Tables

**Table 1:** Demographic and Baseline Clinical Characteristics of the Study Population.

Variable	Responders (*n* = 68)	Non-Responders (*n* = 42)	Total (*n* = 110)	*p*-Value
Age (years)	64.2 ± 10.5	73.1 ± 8.9	68.3 ± 11.2	0.001 *
Female, n (%)	61 (89.7%)	36 (85.7%)	97 (88.2%)	0.542
BMI (kg/m^2^)	29.1 ± 3.6	32.2 ± 4.1	30.4 ± 3.9	0.012 *
Baseline VAS	6.8 ± 0.8	6.6 ± 0.7	6.7 ± 0.8	0.184
Post-procedure VAS	2.4 ± 0.9	5.1 ± 1.2	3.4 ± 1.5	<0.001 *

Abbreviations: BMI, body mass index; VAS, Visual Analog Scale. * Statistically significant (*p* < 0.05).

**Table 2:** Comparison of Radiographic Spinopelvic Alignment Parameters Between Groups.

Parameter	Responders (*n* = 68)	Non-Responders (*n* = 42)	*p*-Value
Lumbar lordosis (°)	45.2 ± 6.4	35.8 ± 7.2	<0.001 *
Pelvic incidence (°)	49.1 ± 8.2	54.4 ± 9.5	0.004 *
Pelvic tilt (°)	17.4 ± 4.5	23.6 ± 5.1	<0.001 *
PI–LL mismatch (°)	3.9 ± 4.2	18.6 ± 7.4	<0.001 *
Sacral slope (°)	31.7 ± 5.2	30.8 ± 6.1	0.412

* Statistically significant (*p* < 0.05).

**Table 3:** Subgroup Analysis: PI–LL Mismatch and Pelvic Tilt.

Subgroup	Responders (*n*)	Non-Responders (*n*)	PI–LL Mismatch (°)	Pelvic Tilt (°)	*p*-Value (PI–LL/PT)
Younger & lower BMI (Age < 65, BMI < 30)	25	12	2.8 ± 3.1	16.1 ± 3.4	0.003/0.008
Older & higher BMI (Age > 65 or BMI > 30)	43	30	4.2 ± 4.8	18.5 ± 5.2	<0.001/0.001

**Table 4:** ROC Analysis for Identification of negative short-term diagnostic response.

Parameter	AUC	95% CI	Cut-Off Value	Sensitivity (%)	Specificity (%)
PI–LL mismatch	0.831	0.754–0.908	>12.5°	84	81
Pelvic tilt (PT)	0.814	0.738–0.890	>21.5°	76	78
Age	0.725	0.615–0.835	>65 years	68	71

Abbreviations: AUC, area under the curve; CI, confidence interval.

**Table 5:** Multivariate Logistic Regression Analysis for Factors Associated With negative short-term diagnostic response.

Variable	Odds Ratio (OR)	95% CI	*p*-Value
PI–LL Mismatch (Continuous, per 5° increase)	1.74	1.28–2.36	<0.001 *
PI–LL mismatch > 12.5° (Categorical)	4.25	2.10–8.60	<0.001 *
Age (Continuous, per year increase)	1.03	0.99–1.07	0.082
Age > 65 years (Categorical)	1.84	0.98–3.45	0.058
BMI > 30 kg/m^2^ (Categorical)	1.42	0.72–2.80	0.312

* Statistically significant (*p* < 0.05).

## Data Availability

The datasets generated and/or analyzed during the current study are available from the corresponding author on reasonable request.

## Abbreviations

CLBP	Chronic Low Back Pain
MBB	Medial Branch Block
PI	Pelvic Incidence
PT	Pelvic Tilt
SS	Sacral Slope
LL	Lumbar Lordosis
PI–LL	Pelvic Incidence—Lumbar Lordosis mismatch
VAS	Visual Analog Scale
ROC	Receiver Operating Characteristic
AUC	Area Under the Curve
CI	Confidence Interval
OR	Odds Ratio
BMI	Body Mass Index
